# Supplementary material for: Are carbon emissions trading and green financial instruments synergistic? -Comprehensive quantitative research based on content analysis
Source: PLoS One. 2024 Mar 7;19(3):e0298601. doi: 10.1371/journal.pone.0298601 (PMC10919601; doi:10.1371/journal.pone.0298601)
Supplement: S1 Data — (ZIP) [file pone.0298601.s001.zip › Data/地级市Z_F工作报告.zip!/╣π╢1⁄2/╖≡╔╜╩╨/└┤╫╘╣1⁄2╓┌║┼í╛┬φ┐╦╩2╛▌═°í┐_2012.docx]

各位代表：

　　现在，我代表佛山市人民政府向大会报告政府工作，请予审议，并请政协各位委员和其他列席人员提出意见。

　　过去五年工作回顾

　　本届政府履职时期，是佛山发展历程中很不平凡的五年，是在困难中奋勇拼搏、在创新中焕发生机的五年。五年来，我们在省委、省政府和市委的坚强领导下，紧紧依靠全市人民，激发全市智慧，全面贯彻落实科学发展观，抢抓《珠江三角洲地区改革发展规划纲要》（下称《规划纲要》）实施等重大机遇，经受国际金融危机等严峻挑战，推动产业转型、城市转型和环境再造取得重大成效，全面完成“十一五”规划各项指标和市十三届人大会议确定的各项目标任务，实现了“十二五”良好开局。

　　五年来，我市经济发展持续跨越，综合实力大幅提升。预计全市地区生产总值、工业总产值、财政总收入全部实现翻番，分别达6600亿元、17800亿元、1111亿元。生产总值五年跨4个千亿元大关，年均增长14.8%；人均生产总值91500元，年均增长10.4%。

　　五年来，我市环境再造不断加强，城市环境明显改善。创建成为国家环境保护模范城市。万元GDP能耗呈逐年下降趋势，主要污染物排放总量控制任务超额完成。

　　五年来，我市民生事业加速发展，人民生活质量不断提高。五年投入民生事业800多亿元，约占全市财政一般预算支出的60%。2011年，预计城镇居民人均可支配收入30705元、农村居民人均纯收入13788元，分别比2006年增长62.5%和67.7%；城乡居民储蓄（本外币）存款余额4729亿元，比2006年增长71.2%。

　　五年来，我们主要推进了以下五方面工作：

　　一、创新发展思路，超前引领经济社会发展

　　创新经济发展思路。出台“精细发展第一产业，优化发展第二产业，加快发展第三产业”发展战略，引导三大产业协调健康发展。实施金融发展三项计划[1]，以金融创新推动产业转型升级。提出“四化融合，智慧佛山”战略，以信息化带动工业化、提升城市化、加快国际化。早谋快断，出台配套政策应对金融危机冲击，经济实现率先突围。

　　创新城市发展思路。实施“强中心”战略，构建“1+2+5+X”[2]组团城市格局。推出城市可经营项目多元化、市场化，解决城市建设资金不足问题。探索以“三旧”改造破解土地瓶颈，开创“佛山模式”。

　　创新政府管理思路。设立市政府专家顾问团，借“外脑”促科学决策。通过举办重大活动和政务公开、网络问政、政务微博等方式，搭建政府与企业、市民交流互动平台。建立健全绩效考核机制,提升科学发展水平和工作效能。

　　二、坚持科学发展，着力推进“两转型一再造”

　　产业转型成效显著。农业精细发展。五年共建现代农业园区17个，新增市级农业龙头企业18家，2011年农地产出率预计达1.9万元/亩。工业优化提升。实施“三个一批”和“双转移”战略，推动传统产业转型升级，五年关停“三高一低”企业约700 家。培育发展战略性新兴产业和先进制造业，引进一批龙头项目，光电显示和光照明产业链日趋完整，汽配产业成为重要经济增长点，机械装备跃升为第一支柱产业。服务业发展提速。金融业集聚效应明显，全国性股份制银行全部落户佛山，广东金融高新区初步建成为现代金融后援服务基地。现代物流业发展迅速，创意设计产业快速崛起，总部经济发展态势良好。招商引资有效促进产业结构调整，五年共引进超千万美元项目332个，总投资175.44亿美元。民营经济继续成为推动我市经济又好又快发展的中坚力量。

　　城市转型步伐加快。实施“强中心”战略，明确佛山新城规划定位和管理体制，一批重点项目和基础设施建设全面提速。五年新增高速公路313.5公里，形成密度全省第二的高速公路网。广佛地铁首通段、广珠城轨佛山段通车，佛山机场民航复航，基本形成现代综合交通体系。实施公交优先发展战略，建立公共自行车系统，市民出行环境明显改善。推进智能交通、智能环保、智能电网、智能警务、数字城管等建设，打造“U-佛山” [3]。第二水源首期工程建成通水，城乡防灾减灾工程基本完成。“创文”取得阶段成果，被中央文明委授予“全国文明城市提名资格”。

　　环境再造成绩突出。实施产业倒逼和退出机制，对陶瓷、纺织、水泥、小火电等行业关停并转。以汾江河为重点的水环境治理成效显著。环保基础设施建设加强，五年新增污水处理能力155.5万吨/日,城镇生活污水处理率达81%。“三年促变、绿地佛山”目标顺利实现。省立、城市绿道全线贯通。顺利通过国家卫生城市和国家园林城市复查。

　　发展要素支撑不断强化。加强金融、科技、人才对产业发展的支持，破解土地、能源瓶颈。着力缓解中小企业融资难题，五年新增上市公司19家、股权投资基金80只、小额贷款公司12家。与中科院、中关村开展全面战略合作。专利、品牌、标准战略实施成效显著，成为国家知识产权工作示范城市、国家商标战略实施示范城市。通过完善人才落户等政策，让各类人才扎根佛山；通过“三旧”改造腾出土地优先发展工业和现代服务业；通过落实电网投资、加强有序用电和节能降耗，电力紧缺局面有效缓解。

　　三、坚持以人为本，大力发展社会民生事业

　　就业和社会保障体系日臻完善。实施城乡统筹的积极就业政策，五年新增就业人数81.6万人，城镇登记失业率控制在3.5%。建立覆盖城乡、功能完善、保障形式多样化的社保体系，五大险种实现市级统筹。健全社会救助体系，救助管理水平不断提高。

　　文教体卫事业全面进步。公共文化服务网络不断完善，重大文化设施建设加强，“魅力佛山”和旅游文化节成为特色文化品牌，祖庙完成百年大修。城乡九年义务教育全面免费，各类教育均衡发展，成为全省首个推进教育现代化建设先进区域、教育综合改革试验市，国家级教育信息化整体应用实践与创新公共服务试点市。成功协办广州亚运会。全面推行平价门诊、惠民产房，推动“六有一共享”[4]便民服务，市民“看病难”、“看病贵”问题得到缓解。

　　社会大局和谐稳定。深入开展严打整治和综治大行动，建设综治信访维稳中心，社会治安防控体系不断完善，社会治安明显好转。“六好”平安和谐社区、“十好”和谐文明村居创建基本完成。建立健全应急管理机制，成功应对各种突发事件。安全生产、产品质量及农产品、食品、药品安全监管得到加强，公共安全形势总体平稳。双拥工作深入开展，七次荣获“全国双拥模范城”称号。汶川水磨重建任务圆满完成，援藏援疆工作扎实开展，扶贫“双到”工作成效显著。

　　民生实事全面完成。每年向市民承诺办好一批民生实事，件件有回应，事事有落实。主要有：完成保障性住房建设任务。解决了全市895户渔民的住房困难问题。基本解决革命老区出行难、饮水难等问题。连续5年调高企业退休人员基本养老金，4次提高城乡低保标准。推行居家养老服务。向全市80周岁以上户籍老人发放高龄津贴。完成中小学校舍安全工程目标任务。对学前教育和重度残疾人托养实行补贴。发放价格补贴，全市困难家庭生活得到基本保障。

　　四、坚持统筹协调，积极推进区域城乡一体化发展

　　广佛同城化初见成效。建立市长联席会议协调机制，出台《广佛同城化发展规划》，启动97个同城化建设重点项目，完成35个。年票互通、交通对接、环境同治及教育、医疗、金融、通信同城取得突破性进展。广佛肇经济圈建设同步推进，55个重点项目有序展开。

　　佛港澳合作得到加强。与港澳在科技业、现代服务业、先进制造业和社会事业等方面开展重点对接。一批港资银行、港资私人诊所、港澳个体工商户进驻佛山。粤港金融科技园、粤港产业创新设计中心等项目加快建设。

　　城乡一体化深入推进。坚持以城带乡、以工哺农，推动城市基础设施向农村延伸、基本公共服务向农村覆盖，初步实现城乡户籍管理、规划建设、社会保障体系、劳动力管理、人居环境建设、义务教育六个一体化，基本实现基层行政服务中心、基层文化服务点全覆盖。首批名镇、名村建设积极推进。

　　五、坚持先行先试，深入推进体制机制改革创新

　　行政体制改革再领风气之先。率先推行市区两级大部制改革，顺德区综合改革成为全省示范。市直事业单位分类改革基本完成。简政强镇事权改革全面铺开，行政审批制度改革不断深化，我市成为省创新行政审批方式试点城市。

　　农村改革不断深化。集体经济管理体制改革稳步推进，90%以上集体经济组织完成“两确权”[5]基础工作，股权固化到户试点进展顺利。“政经分离”、“政社分离”有序推进。引入中介组织规范农村财务管理，农村集体资产管理交易平台投入运行，农村财务网上监控平台完成试点任务。

　　经济体制改革扎实推进。深化部门预算改革，公共财政管理体制日趋完善；适当调整市与区财政体制，有效集中市级财力办大事。实施投融资体制改革，城市可经营项目推介成绩显著，五年吸引投资2097.7亿元。深入推进国资改革，国有企业从一般竞争性领域全面退出。以“大国资、全覆盖”思路，探索国资改革发展新路子。

　　社会体制改革稳步实施。医药卫生体制改革三年重点任务基本完成，社区医疗卫生机构基本实现全覆盖。启动社区管理体制改革试点，社区党组织、居委会、行政服务中心“三位一体”模式得到推广。行业协会实现“民间化”管理，社会组织数量位居全省前列。

　　与此同时，我们高度重视政府自身建设。自觉接受人大和政协监督，认真办理代表建议和委员提案。加强法治社会建设，完成“五五”普法工作。建立领导大接访机制，设立网上信访大厅，开通“12345”行政服务投诉热线，群众诉求得到有效纾解。认真落实党风廉政建设责任制，严肃查处一批违纪违法案件。开展机关作风主题建设活动，政风行风进一步好转。

　　此外，审计、统计、人口计生、民族宗教、外事侨务、对台港澳事务、粮食储备、地质、地震等工作取得新成绩，妇女儿童、老年人、残疾人、广播电视、新闻出版、人防、气象、档案、方志等事业有新发展。

　　各位代表，过去五年，我们在科学发展的道路上迈出了坚实步伐，取得了显著成绩。这一切无不凝聚着全市人民的智慧和汗水，无不饱含着社会各界的关心和支持。在此，我代表佛山市人民政府向全体市民，外来务工人员，中央、省驻佛山单位，驻佛山人民解放军指战员、武警官兵和社会各界人士表示崇高的敬意，向长期关心支持佛山建设的港澳台同胞、海外侨胞、国际友人表示衷心的感谢！

　　我们同时清醒地看到，我市经济社会发展仍然存在一些困难和问题，主要是：发展方式比较粗放，自主创新能力不强，现代服务业发展滞后，经济发展的质量和效益有待提高；土地、能源、环境等约束日益突出，节能减排任务艰巨；城市建设管理相对滞后，统筹区域发展力度有待加强；社会治安、安全生产、劳资关系等方面还存在一些隐忧；政府部门的公信力和执行力有待进一步增强。对此，我们将采取有效措施，努力加以解决。

　　今后五年奋斗目标及2012年主要任务

　　未来五年政府工作的指导思想是：以邓小平理论和“三个代表”重要思想为指导，以科学发展观统领经济社会发展全局，贯彻落实《规划纲要》和市第十一次党代会精神，围绕建设“先进制造基地、产业服务中心、岭南文化名城、美丽幸福家园”的目标，以争创全国文明城市为抓手，继续解放思想，大胆改革创新，加速转型升级，提升城市品质，加强社会建设，努力惠及民生，全面增强城市综合实力和核心竞争力，奋力开创“民富市强、幸福佛山”建设新局面，争当“加快转型升级，建设幸福广东”的排头兵。

　　未来五年经济社会发展目标是：全市地区生产总值力争年均增长10％，突破万亿元，人均生产总值超2万美元；万元GDP能耗和主要污染物减排完成省下达指标；经济规模争取进入全国前十名，力争成为广东最大的先进制造业城市，辐射华南的产业服务中心；“四化融合，智慧佛山”规划重点任务基本完成，社会生产力和城市管理水平大幅提升；实现“学有优教、病有良医、老有善养、住有宜居、行有通衢、治以法尊”的社会发展目标。

　　未来五年，推动我市又好又快发展，需要把握好“五个重点”，力争取得“三个显著成效”。“五个重点”：把加快转变经济发展方式作为工作主线。围绕产业结构调整优化升级，着眼于速度、质量、效益相统一，人口、资源、环境相协调，大力发展先进制造业、战略性新兴产业和现代服务业，着力完善产业链，促进经济增长由粗放型向集约型转变。把增强创新能力作为发展重要支撑。以更大力度鼓励支持创新，使创新成为我市发展主旋律和原动力。着力打造创新平台和高端载体，加大创新研发投入，发挥企业在创新中的主体作用，促进创新思维持续迸发、创新成果不断涌现。把破解资源能源等要素瓶颈作为发展重要抓手。树立科学的要素利用观，以创新举措破解要素困局，以促进内生增长减低要素依赖。把改革创新作为工作致胜法宝。持续保持改革创新优势和活力，以更大决心、更高视野破除各种思想和体制机制障碍，以改革促发展提速，以创新促工作提升。把发展成果普惠共享作为发展根本。坚持城乡统筹发展，公共财政安排侧重于社会发展和民生改善，推动基本公共服务均等化。把人民群众利益放在工作首位，着力解决人民群众最关心、最直接、最现实的利益问题，让人民群众从不断发展中得到更多实惠。“三个显著成效”：构建现代产业体系取得显著成效。先进制造业、新兴产业、高技术产业快速发展，服务业比重和水平逐年提高。到2016年，力争先进制造业、高技术产业产值占工业总产值比重分别达33%和8%，年均增长分别为19%和12%；服务业占GDP比重40%，年均增长15 %。城市升级取得显著成效。建成一批重大交通基础设施及城市标志性建筑，基本完成城市干道、旧城区整治和生态景观林带建设，城市面貌焕然一新。到2016年，新增城市绿地1200公顷，城市绿化覆盖率达40%，水环境、空气质量显著提升。幸福佛山建设取得显著成效。民生事业大幅改善，市民收入逐年增加，社会保持和谐稳定，市民幸福感满意度稳步提高，力争2014年创建成为全国文明城市。

　　2012年是实施“十二五”规划的关键之年，也是新一届政府的开局之年。今年宏观经济运行面临复杂形势，世界经济复苏的不稳定不确定性上升，国内经济增长的下行压力增大，这是佛山与其他城市一样面临的形势。但我市有工业基础好、民营经济强、体制机制活、基层发展动力足等优势，我们完全有条件发展得更好一些、更快一些。只要我们认清形势，坚定信心，全面贯彻落实中央经济工作会议和省委十届十一次全会精神，稳中求进，统筹做好稳增长、促和谐、调结构、惠民生等各项工作，我市经济社会一定能实现率先发展、科学发展。

　　今年我市经济社会发展主要预期目标是：地区生产总值增长10%；人均生产总值增长8.5%；单位GDP能耗下降、主要污染物减排量均完成省下达任务；地方财政一般预算收入增长10%；居民消费价格总水平涨幅控制在4%左右。

　　实现上述目标任务，我们要突出抓好以下六方面工作：

　　一、加快产业转型升级，着力提升经济发展质量和效益

　　全力构建现代产业体系。发展现代农业。加强现代标准农田和设施农业建设，强化农业科技支撑引领作用，建设一批农业生态园区和菜篮子基地，发展一批农业龙头企业和农民专业合作社。推动制造业高级化。制定制造业转型升级促进质量和效益“双提升”计划，积极推进商业模式创新和科技创新公共平台建设，推动传统优势产业转型升级和品牌化发展。加速信息化与工业化深度融合，鼓励企业采用物联网、ERP[6]、CAD[7]等信息技术，提高产品设计开发能力，规范提升企业综合管理水平，推动产业高端化发展。立足现有产业优势，培育引进一批先进制造业和战略性新兴产业，推动规模化、集群化发展。深入实施专利、标准、品牌战略，鼓励企业加大研发投入和品牌营销，着力推动产业向“价值微笑曲线”两端延伸。制定优惠扶持政策，充分发挥传统优势产业和光电、新能源汽车等龙头企业带动作用，着力完善上下游产业配套和服务能力，打造全产业链核心竞争力。发展壮大现代服务业。全力落实现代服务业发展“314计划”[8],积极推动制造业主辅分离试点，抓紧实施“智慧物流腾飞计划”[9]，出台鼓励总部经济发展的政策措施，加强佛港澳现代服务业领域深度合作，提升高端服务业水平，力争第三产业比重有明显提高。

　　促进产业、科技、文化、金融融合发展。深化与中科院、中关村的全方位多层面战略合作，推动科技成果在佛山转化为现实生产力。大力推进中国南方智谷建设，依托科技园区、专业镇、产业基地，建设和完善面向中小企业的科技创新服务体系，提供关键核心技术攻关、产品研发、质量检测认证、职业教育培训等服务。高度重视文化艺术对产业发展的推动作用，引导企业通过工业设计和文化创意提升产品品质、创造市场需求。落实各项人才政策，优化创新创业环境，完善人才配套服务，大力培养和引进高技能人才、创新团队和领军人物。继续推动企业上市发展。积极争取国家和省的金融创新政策，实施金融“聚变”计划，引入国内外各类金融机构、股权投资基金，引导动员民间资本参与设立本地金融机构，着力构建服务于中小微型企业融资发展的新型金融体系，使金融成为助推产业转型升级的核动力。

　　提升招商引资质量和水平。建立招商项目科学评估体系，明确将投入产出率、税收贡献、能耗、环境要求等作为引进项目的重要评价指标，既注重引进带动能力强的龙头大项目，又注重引进用地少、科技含量高、效益好的项目。实施产业链招商行动计划，对现有行业产业链进行调查分析，确定招商重点区域，排出招商主攻目标，突出大项目，加强产业链招商、以商引商、科技招商和增资扩产，促使产业链的上下游客商来佛山投资建厂，形成产业集聚发展，实现招商引资倍增效应。

　　打造高端产业发展载体。着力提升佛山高新区承载高端产业的能力，调整优化资源，规划打造一个具有核心园区、独立财政、充分授权、环境优美、服务完善的全国一流的国家级高新区。加强佛山新城、广东金融高新区、广东工业设计城、华南智慧新城、国家环境服务业华南集聚区等现代服务业载体建设，着力打造广东新光源产业基地、广东太阳能产业基地、广东新材料产业基地、广东物联网应用产业基地、广东新能源汽车产业基地，为新兴产业发展提供高端载体。将“三旧”改造与城市升级、产业转型紧密结合，鼓励引导旧厂房、低效工业区整体改造、连片开发，建设一批专业园区。大力支持顺德清远（英德）经济合作区建设，努力打造成新型区域合作示范区。坚持电力设施先行，“十二五”时期投资超过200亿元，加快建设“7+6分区互联”现代化电网[10]，支撑和保障我市经济社会发展。

　　培养壮大企业家队伍。着力为企业家成长提供政策、搭建平台、优化环境、提升服务。出台“企业家成长促进计划”，通过事业、物质、精神等激励，引导行业领军人物、优秀企业家茁壮成长。加强工业园区、专业镇、特色产业基地以及专业孵化器建设，为企业家成长搭建良好创新创业平台。鼓励和支持企业家“走出去”，开拓视野，提升能力水平，把企业做强做大。满腔热情、想方设法帮助企业解决土地、用电、资金、人才等方面的实际需求和困难，在全社会营造鼓励创新创业、宽容失败的社会氛围。

　　二、加强城市建设管理，全面提升城市品质

　　以佛山新城建设携领组团城市发展。加快推动佛山新城崛起、桂城千灯湖片区拓展和禅城老城区改造提升，共同支撑“强中心”战略实施。全力做好佛山新城土地利用总体规划调整，狠抓重点项目和基础设施建设，融合生态、文化、智慧等要素，突出在“水环境”和“建筑风格”上下功夫，把佛山新城建成具有岭南风韵的城市精品。各区城市建设要突出重点，禅城加快祖庙东华里、东平河北岸片区建设；南海推进沥桂一体、三山新城、狮山中心城区、听音湖片区建设；顺德以建设顺德新城、滨水魅力小城和国际生态谷为重点，打造网络型城市典范；高明推进西江新城建设；三水加强三水新城建设。同时，积极推进城镇化建设，着力打造一批产业与生态相兼容、快节奏与慢生活相结合的富有岭南特色的优美镇（街），加快形成“1+2+5+X”城市发展格局。

　　加快交通基础设施建设。加强全市交通规划统筹，以TOD理念[11]打造公交都市[12]，以公共交通引导城市发展。加强轨道交通规划建设，抓紧启动广佛线二期建设，科学规划地铁2号、3号线路线，做好沿线土地规划控制，推进地下空间开发利用，建立土地开发与轨道交通建设捆绑机制。加快在建高速公路项目建设，今年力争完成佛开高速扩建、广三高速扩建、广明高速二期建设任务。加紧樵高路，魁奇路东、西延线，佛陈大桥扩建等项目建设，抓好原325、321国道迁移和改造提升及新国道的标志、标线设置和快速化改造，打通市域交通瓶颈。支持南海新型公共交通系统[13]建设，总结经验，统筹推进各区新型公共交通系统建设，并纳入全市公共交通票制系统。坚持公交优先，促进公交与地铁、有轨电车等各种交通系统的衔接，提升公共交通整体服务水平。规划建设现代化停车系统，解决“停车难”问题。

　　实施城市升级三年行动计划。以重点项目为抓手，以监督考核为保障，做到规划、资金、土地、时间进度和责任单位“五落实”。围绕城市核心区、城市主要轴线、重要滨水地区、门户地区及特色步行街实施升级计划，突出加强铁路、高速公路、主干道路、河涌两侧土地控制和生态景观林、绿化景观带建设，对重点区段进行建筑立面、人行道、路灯、路面、绿化等“五位一体”改造，对城际交界面和主要出入口进行生态绿化和景观提升，构建组团间生态绿道连接系统和绿廊隔离系统。深化汾江河综合整治，大力推进大墩村河涌、南北大涌、谢边涌、乐平涌等环境治理，加强工业锅炉、VOC[14] 专项整治，加快淘汰黄标车，严厉打击环境违法行为，持续改善大气和水环境。把实施城市升级三年行动计划与创建全国文明城市、全国绿化模范城市、国家生态市结合起来，实现城市环境、形象、内涵和品质的全面提升。

　　加强智慧城市建设。建立城市公共资源综合利用体系，统筹规划和建设水、电、煤、气、通讯等综合管网，提高土地利用效能。出台智慧佛山新城规划，重点推进智能交通、智能电网和智能社区建设。华南智慧新城着力打造全国智能产业标兵。千灯湖金融智慧新区重点推进无线城市建设，加快通讯、电力、交通等基础设施的高度智能化。顺德德胜总部商务区建设高品位的智慧城市综合体。实施光纤入户工程，构建高效便捷信息网络体系，建设光网城市。完善电子政务数据中心建设，统筹全市政务数据资源，加快推进智能交通、智能城管、智能教育、智能卫生、智能文化、智能社保、智能警务等工程建设，构建服务型智慧政府。

　　积极融入珠三角一体化发展。抓住广州南沙、深圳前海、珠海横琴开发机遇，主动对接，借势发展，融入珠三角一体化进程。认真落实《规划纲要》“四年大发展”实施方案，力争完成85个重点项目建设任务，为“十年大跨越”奠定坚实基础。进一步提升广佛同城化建设水平，继续推进广佛环线佛山西站至广州南站轨道交通、珠江大桥放射线接广佛新干线、龙溪大道接海八路快速化改造、佛陈路东延线接番禺双涌路（海华大桥）、金沙洲地区市政道路桥梁对接等一批重点交通设施对接。积极推动重点区域合作，完成五沙地区、花都空港地区同城化整合规划，推动与广州南站核心区深度融合发展，探索开展金沙洲地区社会管理合作。加强民生事业合作，今年力争实现通信同城，逐步推进金融同城，促进科技教育、医疗卫生、社会管理和公共服务合作取得更大成效。推动广佛肇经济圈建设，落实年度重点合作项目，探索共建经济合作区。

　　三、加强和创新社会管理，建设幸福美好生活

　　创新社会服务管理。充分发挥政府主导作用，大力培育和扶持发展公益慈善类、社会服务类、行业协（商）会等社会组织，在创新社会服务管理中注重发挥各类社会组织的积极协同作用、工青妇组织的桥梁纽带作用。加强社区、村居建设，实现基层民主自治与政府行政管理有机结合，引导人民群众平等参与自治和管理，让人民群众成为社会管理和服务创新的主人。完善社会自愿服务体系，推广南海“关爱桂城”、顺德“两社三工”[15]模式，形成党委领导、政府负责、社会协同、公众参与的社会管理格局。创新流动人口服务管理，促进流动人口基本公共服务均等化。

　　加快发展社会事业。促进各类教育优质均衡协调发展，实施《学前教育三年行动计划》，支持佛科院向教学与研究并重型综合大学发展，稳步推进教育综合改革试点工作，提升教育现代化发展水平。继续推行国家基本药物制度，完善社区卫生服务网络和医疗便民惠民措施，加强基层卫生队伍建设，强化疾病预防控制和公共卫生保障，为市民提供优质医疗卫生服务。创新人口服务与管理机制，稳定低生育水平。

　　促进就业和完善社会保障体系。落实各种就业优惠政策，加强就业技能培训，拓宽就业渠道，大力开发公益性岗位，鼓励和支持自主创业，全力做好城镇就业困难人员、农村转移劳动力、高校毕业生等就业安置工作，解决结构性失业问题。继续突出社会保险扩面工作，加大基金征缴、稽核、清欠等管理力度。完善生育保险、新农保、城镇居民社会养老保险等城乡一体化社会保障体系。

　　切实做好综治维稳工作。深入开展“平安村居”、“民主法治村”和“智能小区”创建活动，加大对各类违法犯罪活动打击力度，确保群众生命财产安全。健全综治信访维稳机制，完善政务微博和网络问政平台，畅通群众诉求渠道，增强社会矛盾调处能力。加强“六五”普法工作，大力弘扬法治精神，引导群众以法律手段化解矛盾纠纷。确立安全发展理念，强化安全生产、消防和农产品、食品、药品安全监管，依法打击违法违规行为。完善应急救援机制，预防和处置好各类突发公共事件。

　　办好一批民生实事。继续加大财政投入，为市民多办实事、多办好事。加快保障性住房建设，今年基本解决我市现有在册城镇低收入困难家庭的住房问题。积极落实稳价惠民措施，推进和完善平价商店建设，完善价格补贴联动机制。加强饮用水源保护，完成9个省级饮用水源保护区标准化建设。稳步推进城市生活垃圾分类试点及餐厨垃圾处置工作，力争今年城镇生活垃圾无害化处理率超过89％。在10家社区卫生服务中心开展“健康e园”[16]建设试点，为市民免费提供自助体检服务。建立并完善生育保险制度，将生育医疗费用和生育津贴纳入基本医疗保险保障范围。全面推行国家免费孕前优生健康检查项目。对在本地半年以上、且在本地落实绝育措施的流动人员，发放节育奖。建立残疾人津贴制度，向0~6岁残疾儿童提供免费康复服务，建设佛山市启聪学校残疾学生职业培训、残疾人就业技能培训和重度残疾儿童教养基地，实行特殊教育15年免费教育。加强公共体育设施建设，今年争取每个行政村（社区）都建有户外健身设施，逐步推动有条件的学校体育设施开放共享，建设佛山新城露天沙滩泳场并向市民开放。实施农村电影放映工程，让农村居民就近看到质优价廉电影。继续调整企业退休人员基本养老金。全面落实社区居家养老服务，适度扩大服务对象范围，提高服务标准。启动家庭服务中心[17]建设试点。抓好革命老区扶贫开发“双到”工作，多举措帮助贫困家庭脱贫致富，早日过上幸福生活。

　　四、注重内涵发展，大力提高文化软实力

　　加强社会道德风尚建设。加大社会主义核心价值体系宣传教育力度，大力弘扬新时期佛山人精神，着力提升公民文化道德素养，构建积极向上、和谐康宁的社会心态。坚持“创建为民，五区同创，各方参与”原则，继续推进全国文明城市创建活动，加强信用城市建设，全面提升城市文明程度和市民文明素质，建设和谐社区。积极倡导市民行善举做好人，形成引人向善的良好风尚。加强军民共建，巩固“双拥”成果。

　　提升公共文化建设水平。深化文化体制改革，创新公益性文化事业发展模式，把文化建设纳入科学发展考核评价体系。加强公共文化基础设施建设，高标准建设佛山新城公共文化综合体等一批文化地标。大力开展群众性文化活动，推动文化下基层，高水平办好佛山秋色欢乐节等民俗文化活动，做强做优“魅力佛山”等系列文化品牌，让广大市民充分享受“文化大餐”的滋养。

　　加强历史文化保护传承。深入挖掘佛山文化底蕴，统筹推广全市五区文化精华，总结提炼粤剧、武术、陶艺、香云纱、民间艺术等传统文化精髓，打造城市文化品牌，扩大佛山知名度。抓好名镇名村建设。科学推进旧城区改造，在城市改造和建设中更加注重文化、文物保护。着力培养一批文化艺术大师和民间艺术创作领军人才。

　　加速文化产业发展。积极推动文化资源开发与城市升级、产业发展相融合，重点加快岭南天地等历史文化街区建设，打造特色文化功能区；大力发展工艺美术、创意设计、影视演艺、文化会展、新闻出版、文化休闲娱乐等产业，培育壮大一批文化龙头企业。加强佛山传媒集团、广东香云纱文化产业园、佛山创意产业园、佛山新媒体产业园、39°艺术创意空间等文化重点园区和产业基地建设，促进文化产业集聚发展。打造一批文化旅游品牌，支持西樵山创建国家5A级旅游景区。

　　五、勇于改革创新，进一步增强发展动力和活力

　　完善行政体制改革。深化大部制改革，进一步理顺市、区、镇（街）行政管理运行机制，探索建立定位准确、配置合理、关系顺畅、权责统一的政府职能体系。进一步强化市级在重大规划、重大产业布局、重大事项决策等方面的统筹力度，确保全市协调、均衡、健康发展。抓紧推进联合审批改革，完善以行政审批电子网络一体化为核心的政府“虚拟”服务大厅建设。

　　加强社会体制改革。制定政府职能转移和购买服务制度，推动政府向社会放权。通过设立社会组织发展专项资金、建立社会组织孵化基地、引入竞争机制，促进社会组织更好地承接政府转移的事项。顺德区要抓紧推进社会体制综合改革，成为全省社会建设和管理创新的尖兵。深化住房保障和医疗保障制度等民生领域改革。建立健全以中低收入中住房困难家庭为保障对象、以公租房为供给主体的新型住房保障制度。引入市场化管理方式改革基本医疗保险管理制度，健全基本医疗保险筹资和待遇水平调整制度，积极推进公立医院改革试点。

　　深化农村体制改革。推广南海“政经分离”、顺德“政社分离”模式，推进农村基层管理体制改革，建立农村基层管理协同共治机制和经济发展利益共享机制。创新农村集体资产运营管理模式，探索股权社会化流转。开展农业设施、土地承包经营权、林权等抵押贷款试点，拓宽农业融资渠道。持续扩大公共财政覆盖农村的范围，促进农村全面进步。

　　全面推进财政、国资改革。实施财政综合管理改革，创新政府理财思路、投入机制和管理方式，综合统筹、高效运营、优化配置财政、国土、国资、行政事业单位资产等资源，全面提升政府经济管理水平和效益，逐步构建持续、稳健的政府财政保障体系。推进新一轮国资大改革、大发展，加快国资土地储备分中心建设和国有企业架构重组，进一步盘活国有资本，通过资本运营筹措城市建设、经济发展资金，实现国资与财政“两条腿”走路。

　　六、加强自身建设，努力提高政府执行力和公信力

　　切实提高政府决策水平。探索应用信息技术，建设动态开放政府。主动接受人大、政协、社会团体、舆论和人民群众的监督，坚持民主集中制，完善行政决策规则，严格落实专家咨询、社会听证、决策评估、合法性审查等决策制度，让决策更符合全市发展及市民利益需求。建立决策实施评价和责任追究制度，对违法决策、随意决策行为严肃处理，增强政府公信力。

　　切实提高政府执行力。完善和落实绩效考评机制，提高政府机关效能。健全重点工作任务分解落实机制，明确目标、任务、要求和完成时间，加大检查督办力度，使各项决策和重点工作有人负责、有人落实、有人跟踪、有人督办。敢于直面和克服困难，狠刹遇事推诿、办事拖拉等不良风气，打造执行力强的效能政府。

　　大力加强政府廉政建设。弘扬廉政文化，加强公务员廉政道德培训和考核，营造勤政廉政的良好社会氛围。建立健全政府法制监督系统和综合型、全方位行政电子监察系统，实行网上监控行政权力运行。坚决查处违法违纪案件，提高反腐败效能。

　　大力加强公务员队伍建设。完善公务员选用机制，从农村、社区优秀基层干部和有基层经历人员中考录公务员。健全竞争性选人用人机制，探索公务员与企业干部双向交流制度，推进公务员跨区、跨部门交流，有计划选派具有培养潜力的年轻干部到基层挂职锻炼，不拘一格，任人唯贤！

　　各位代表！时势日新，催人奋进；时不我待，只争朝夕。让我们高举科学发展旗帜，在市委的正确领导下，更加团结振奋，更加创新进取，更加务实有为，为建设“民富市强、幸福佛山”，创造佛山更加美好的明天而努力奋斗！
